# A hierarchical detection method in external communication for self-driving vehicles based on TDMA

**DOI:** 10.1371/journal.pone.0188760

**Published:** 2018-01-09

**Authors:** Khattab M. Ali Alheeti, Muzhir Shaban Al-ani, Klaus McDonald-Maier

**Affiliations:** 1 School of Computer Sciences and Electronic Engineering University of Essex, Colchester, United Kingdom; 2 University of Anbar, College of Computers-Anbar, Iraq; 3 Department of Computer Science – College of Science and Technology, - University of Human Development – KRG - Iraq; Victoria University, AUSTRALIA

## Abstract

Security is considered a major challenge for self-driving and semi self-driving vehicles. These vehicles depend heavily on communications to predict and sense their external environment used in their motion. They use a type of ad hoc network termed Vehicular ad hoc networks (VANETs). Unfortunately, VANETs are potentially exposed to many attacks on network and application level. This paper, proposes a new intrusion detection system to protect the communication system of self-driving cars; utilising a combination of hierarchical models based on clusters and log parameters. This security system is designed to detect Sybil and Wormhole attacks in highway usage scenarios. It is based on clusters, utilising Time Division Multiple Access (TDMA) to overcome some of the obstacles of VANETs such as high density, high mobility and bandwidth limitations in exchanging messages. This makes the security system more efficient, accurate and capable of real time detection and quick in identification of malicious behaviour in VANETs. In this scheme, each vehicle log calculates and stores different parameter values after receiving the cooperative awareness messages from nearby vehicles. The vehicles exchange their log data and determine the difference between the parameters, which is utilised to detect Sybil attacks and Wormhole attacks. In order to realize efficient and effective intrusion detection system, we use the well-known network simulator (ns-2) to verify the performance of the security system. Simulation results indicate that the security system can achieve high detection rates and effectively detect anomalies with low rate of false alarms.

## 1. Introduction

Self-driving vehicles are rapidly becoming a key autonomous systems technology. They can make a direct and positive contribution to our society by potentially reducing the number of accidents, cost and environmental impact of cars [1]. Autonomous vehicles attempt to replace humans by automating driving to reduce the number of fatalities and injuries on busy roads caused by human errors. Self-driving vehicles rely on ad hoc networks, specifically so-called vehicular ad hoc networks (VANETs). These networks enable flexible communication more flexible between vehicles within the radio coverage area.

Such vehicles rely heavily on data that is exchanged between the vehicles and dedicated infrastructure, i.e. so-called road side units (RSUs). Vehicles cannot move or predict the external environment without data and control from beacons. To achieve this beacons periodically broadcast data from one vehicle to others in that zone. In this case, vehicles accept to receive data from beacons based on specific rules that are installed on vehicles or RSUs [2]. These rules are based on vehicle motions states such as speed, heading, position and time.

VANETs have some characteristics which can be the cause of security problems such as the absence of a fixed security system, open medium wireless communication, speed and a highly dynamic topology [3]. These characteristics expose self-driving and semi self-driving vehicles potentially to many types of attacks, e.g. Sybil, black hole, grey hole, Wormhole and Denial of Service (DoS) attacks [3].

This paper presents the design of an Intrusion Detection System (IDS) to secure the external communication in self-driving vehicles. The proposed system periodically logs, calculates and stores different parameter values from neighboring vehicles to detect both abnormal or malicious behaviour in the network.

Recently, many scholars proposed different types of encryption and authentication algorithms and Finite State Machines (FSMs) to obtain secure communication and routing protocols such as SAODV and ARAN [4], but however, these types of secure routing protocols do not have the ability to resist internal attacks, where the attacker is already based in the VANET, such as Sybil attack. This has motivated the design of a hierarchical IDS to secure VANETs presented here.

Our proposed system is based on parameters which describe the behaviour of vehicles, classified in either normal or abnormal behaviour. To achieve efficient performance, the proposed IDS approach enables us to collect and monitor neighboring vehicles. In other words, we attempt to build a clustering approach that increases the system accuracy and speed, while providing a low false alarm rates in online detection. In order to achieve clustering, we employ a clustering protocol that is based on Time Division Multiple Access (TDMA) [5].

TDMA is used to provide channel access which sharing medium networks based on split signal between nodes in that zone. It divides the signal between users’ networks into different time slots.

**Fig 1:**
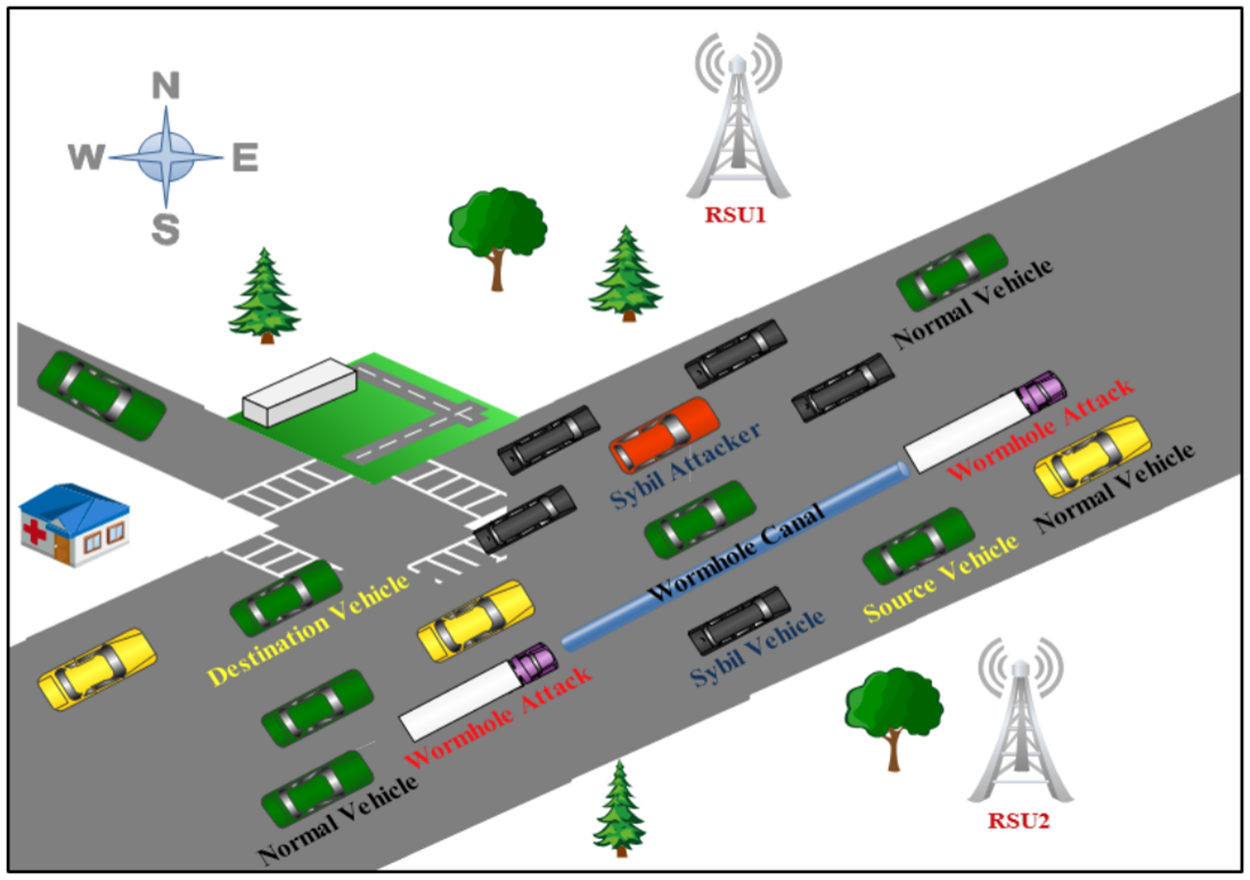
A typical Sybil and Wormhole attacks in VANETs.

Here, an IDS is proposed that can detect vehicles that seek to attack via Sybil and Wormhole attacks to protect the external communication systems of autonomous vehicles. TDMA cluster-based media access control is employed in design IDS to secure the VANETs for vehicles. To achieve stability and maximize channel utilisation, a cluster technique is beneficial for VANETs. TDMA divides signals into time frames and then into time slots, where each vehicle is associated a time slot in the frame [6]. The proposed system has the ability to identify and analyse the positions and identifications of vehicles to calculate distance and angle degree based on this cluster—TDMA scheme. This can be utilised to determine a malicious vehicle behaviour in the wireless network area, Fig 1 shows behaviour of Sybil and Wormhole attacks in the external communication system of autonomous vehicles.

The paper is organized as follows: Section II provides at literature survey of intrusion prevention security systems in self-driving and semi self-driving vehicles. Section III describes intrusion detection in ad hoc networks and the clustering mechanism. Section IV details the design methodology section V provides simulation results and analysis. Section VII discusses outcomes of the scheme and while section VIII concludes and provides a summary of future works.

## 2. Literature survey

The objective of self-driving vehicles is to provide comfort, safety and convenience to road users. In this section, we identify some previous work that is directly and indirect related to our research. Many previous works to secure VANETs were based on IDS; however but many attackers were able to identify weak points that could exploited. Hence VANETs still suffer from a many security problems [7].

Coussement et al. have presented a decision support system based on a clustering approach to protect external communication in vehicles [8]. The authors have installed two IDSs, i.e. on vehicles and other on the RSUs. The IDS on vehicles forward received packets to the cluster members (CMs), neighbouring clusters and to the RSUs for improved detection of attackers. The IDS then generates alarms to notify vehicles and RSUs, to prevent and isolate the attacks attempting to access the resource network. Khan et al. have proposed an algorithm to enhance a Detection of Malicious Nodes (DMV) algorithm based on DMN-Detection of Malicious Nodes in VANETs [9].

The proposed algorithm improves network rates and network performance in detection of attacks by enhancing rate of verifiers. Gover et al. have proposed a novel security system to protect VANETs from Sybil attack [10]. The proposed security system is based on distance and angle parameters to detect malicious vehicles. The system was installed on RSUs to screen all passing vehicles and provided a high detection rate of 99 with low false positive rate of 0.5. Ali et al. have presented an intelligent security system to protect external communication of self-driving vehicles [11]. The proposed system had the ability to detect black hole attacks. The system is based on data that has been collected from a trace file. This trace file was generated from the network simulator, it had high detection rate with low false alarms.

Bruno et al. explained an overview of researches related to intrusion detection concerned with Internet of Things (IoT), which shares many similarities to the VANET environments. They classified intrusion detection systems according to security, threat, validation, methods, and algorithms. In addition, they implemented their approach for IoT environments [12]. Macedo et al. detailed an advanced time division multiple access based on wireless sensor network. This approach aimed toincrease the lifetime of networks supporting alarm-driven applications of delay-sensitive systems. Also, they implemented performance evaluation of latency energy minimization medium access technology. In addition the contributed a compensation for energy consumption and to minimise interference [13].

Vineeth V.V. et al. considered phasor measurement units as a sensor node when data is exchanged between these nodes. They implemented intrusion detection approach for packet loss avoidance using time division multiple access protocols and evaluated the improvement of system security. Attack detection algorithm is implemented via network simulator ns-2 [14]. Tian et al. studied the 2D k-barrier concepts, then applied these concepts on a square region with sensors local neighbor information via applied a distributed scheme. The implemented scheme aimed to lower detection delay and low energy consumption. The barriers constructed in both directions improve the detection of crossing path intruder. This approach is applied on the environment of the network simulator ns-2 in which showed the mechanism in both detection delay and energy consumption [15].

Guo et al. explained that dynamic network topology and variation of routing protocols make it difficult to detect intrusions in wireless sensor networks. This approach implemented a routing protocol based on process algebra applied on wireless mesh networks. In addition, detection of the attack type is implemented via attack points. The strength of this approach is based on the combination of attack points with the implemented protocol in high detection accuracy [16].

Al-Yaseen et al. implemented an adaptive system based on real-time multi-agent system, which is used for unknown attacks in real-time, via an adaptive algorithm based both support vector machine and extreme learning machines. This approach improved the accuracy of intrusion detection and speed up the detection process [17].

## 3. Intrusion detection in Ad hoc networks

Self-driving vehicle networks have some characteristics which make the design of IDS complex. As previously identified above, VANETs are much more susceptible to attacks than other networks because of their dynamic topology, open medium communication and absence of centralized security system [3].

The adoption of both authentication and encryption schemes are considered a strong first layer of defence whilst IDS can form a second layer of defence against intruders [17]. Intrusion prevention can be utilised to enhance the defence capability of such networks, but these mechanisms often suffer from drawbacks such as high costs of implementation and limited coverage to prevent attacks [18]. Thus the nature of communications in self-driving vehicles requires technologies such as intrusion detection (IDS) that are integrated with monitoring and tracking systems to detect any abnormal behaviour.

IDS can log, collect and analyse data that was extracted from VANETs. The IDS then identifies normal or abnormal behaviour and flags four types of alerts i.e. True Positive, True Negative, False Positive and False Negative.

The detection systems in IDS are divided into three types: signature-based, anomaly-based and specification-based detection The anomaly-based IDS divided into two further types which are: self-learning and programmed. Here, we utilise IDS based on data traffic that has been collected from VANETs, and the detection is anomaly-based, with a function that can decide what will be the normal behaviour.

In addition, IDS can be divided into three types based on their architectures, i.e. there are Hierarchical, Cooperative and Stand-alone IDS architectures. Each IDS architecture has related strengths and weaknesses. In our paper, we used a hierarchical architecture IDS due to it significant capability in detecting malicious activities. The strengths and weaknesses of hierarchical architecture are as shown in Table 1:

**Table 1:** Strengths and weakness of hierarchical IDS.

IDS Architecture	Strenghts	Weaknesses
Cluster-based IDS Architecture	High accuracy detection	Vulnerable to attacks
Cluster-based IDS Architecture	Reduce the False Alarms	Point of failure
Cluster-based IDS Architecture	Flexibility	Unfairly overloaded

Traditional intrusion detection techniques based on auditing data and information can describe the behaviour of vehicles. The highly dynamic topology of VANETs has a direct and negative on performance of IDS, which consequently creates challenges for IDS in collecting and analysing data [19]. In this case, we design a hierarchical IDS which can overcome dynamic environments through installing the IDS on cluster head (CH).

## 4. Clustering mechanism

**Fig 2:**
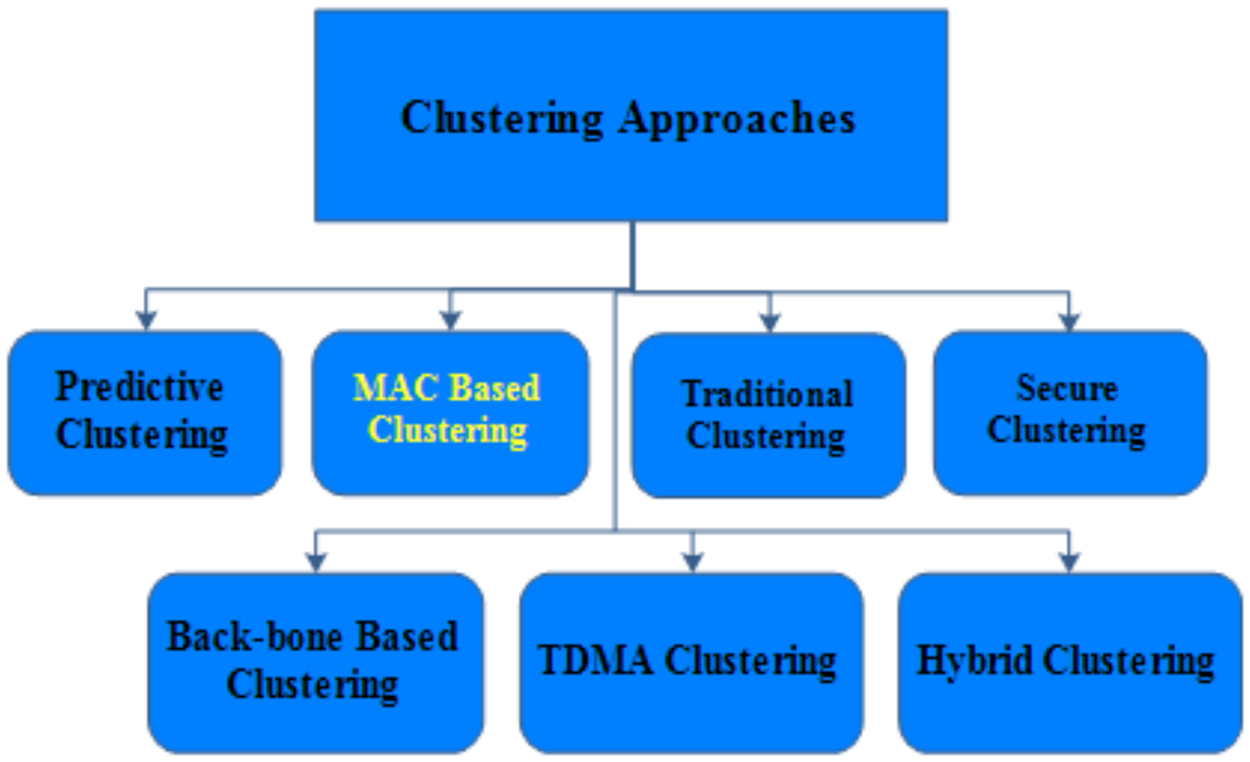
Taxonomy clustering scheme.

In wired networks, we can build an IDS on a centralized authority however wireless networks lack such fixed security. This is encouraging researchers to create virtual centralized or semi-centralized authority by incorporating clustering [6]. Clustering based TDMA architectures provides external communication in self-driving vehicles, whilst offering scalability and fault tolerance resulting in efficient use of VANET resources. Fig 2 shows the taxonomy of existing clustering scheme for VANETs [6].

**Fig 3:**
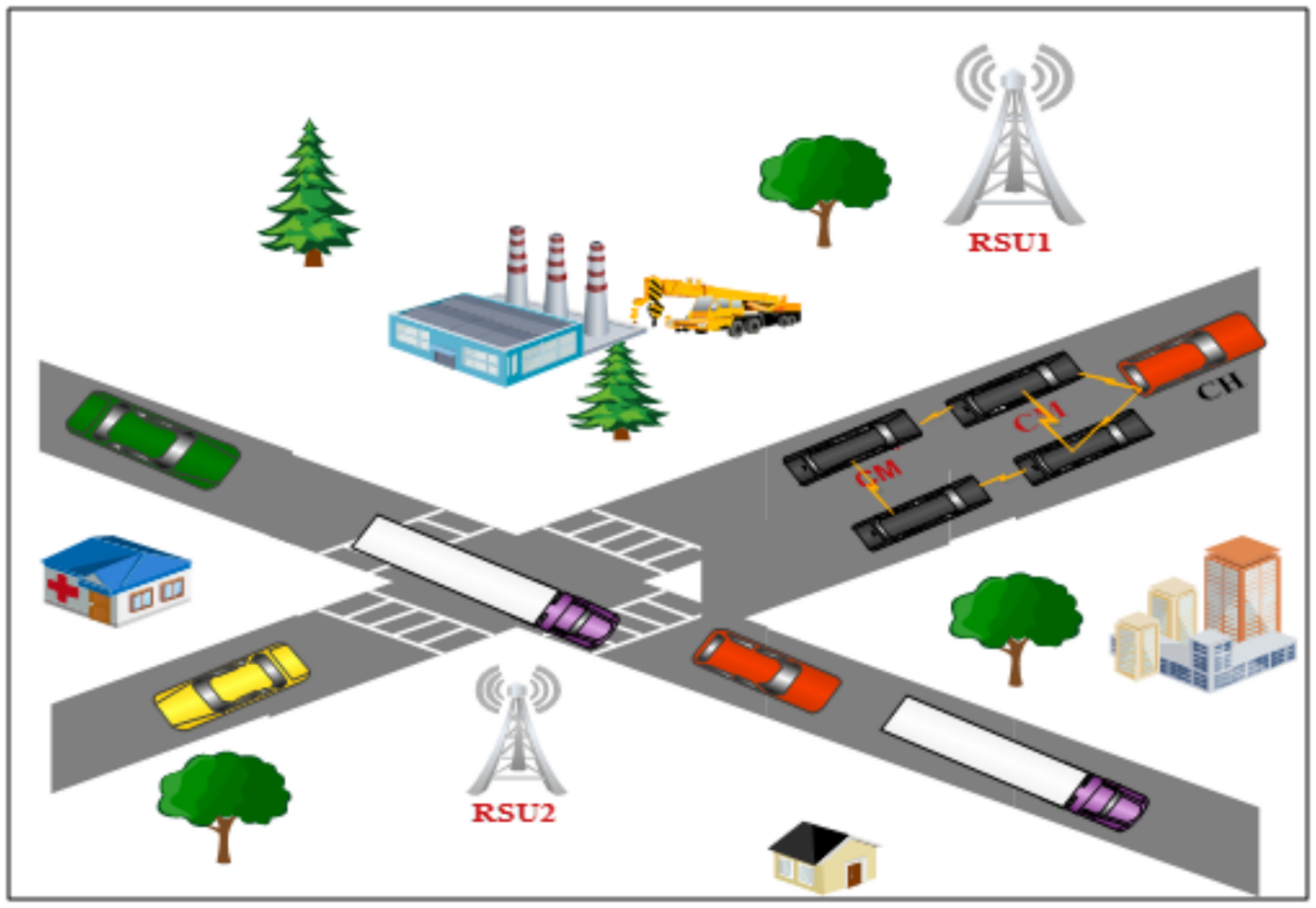
An example of the clustering in VANETs.

The CH receives data traffic and control data from cluster members (CMs) to validate malicious behaviour and generate alarms. The selection of the CH is based on an election algorithms which are utilised in the clusters [6]. The semi centralization optimizes communication between vehicles and vehicles with RSUs. MAC based clustering (TDMA clustering) is used in this paper to achieve stability and maximize channel utilization, a cluster technique is beneficial for VANETs. A MAC algorithm used in the TDMA method allows to decrease the number of packet drops as well as collisions and enables vehicles to transmit on the same frequency channel by using clustering of vehicles. Fig 3 shows the clustering scheme in VANETs.

### A. Time Division Multiple Access (TDMA)

**Fig 4:**
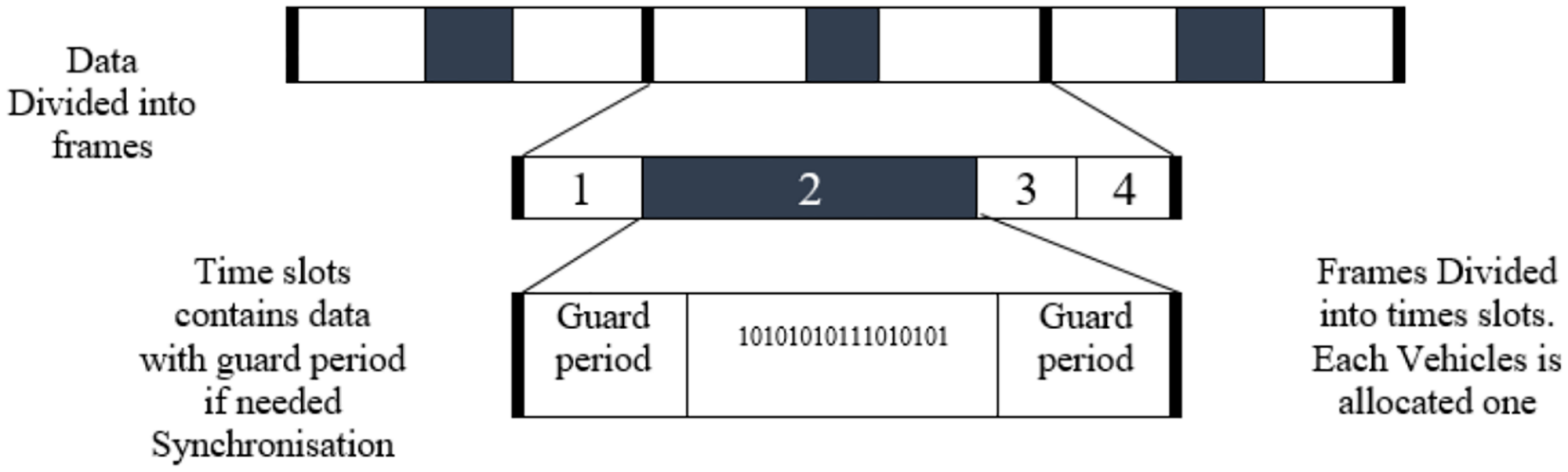
TDMA structure.

TDMA is used to control channel access between vehicles by sharing medium communication based on split signal between nodes in that zone. It divides the signal between users’ by allocating different time slots. Here we design the IDS on clustering head (CH) vehicles. The security system uses the TDMA cluster-based media access control to secure the external communication for self-driving and semi self-driving cars. To achieve stability and channel utilization, the cluster is needed in VANETs. The TDMA divides signal into time frames and it divides the time frame into time slots, where each vehicle is associated with time slot in the frame [6]. Fig 4 shows the working of TDMA.

In addition, TDMA can offer fairness in the sharing of communication channels between vehicles without employing any extra infrastructure or virtual leader vehicle [6]. To design a robust security system, two important challenges are incorporated: aspects, i.e. fuzzy logic and clustering based TDMA.

## 5. Methodology

Autonomous cars’ utilisation of open wireless medium and shared broadcast on wireless channels establishes new security challenges in the external communication systems of these cars. The steps below explain the methodology for proposed security system, namely:

### A. Simulation of highway mobility scenario

To create a real communication environment of autonomous and semi-autonomous vehicles on streets, two tools are utilised to generate traffic and mobility scenarios for these cars. This software is: Simulation of Urban Mobility Model (SUMO) and MObility VEhicles (MOVE) and are commonly employed to generate vehicles scenario [20]. The SUMO software is utilised to simulate highway mobility because it is computationally efficient, flexible and utilises a high number of traffic vehicles [20]. The highway mobility model is employed to evaluate/test the proposed security performance on Highways/streets. The proposed IDS in this paper has the ability to detect two types of scenarios are normal and abnormal behaviour.

### B. Fuzzy membership

Fuzzification technique is utilised in fix one of the common problem of classification which is ambiguity between normal and abnormal behaviours. In more details, sometime border between normal and malicious connection is not sufficient clear [21]. The extracted features from trace file of ns-2 play direct role on the proposed IDS performance [22]. The detection rate will reduce when the name of class whether normal or malicious is not well separated. In addition, the false alarm also will increase that has direct and negative impact on IDS performance. The lightweight of mathematical model of fuzzification is considered a suitable scheme to expose this type of classification problem [21]. Thus, it plays important role in creating clear border within extracted dataset.

$$\begin{array}{c} f(x,a,b,c)=max(min(x-a/b-a),c-x/c-b),0) \end{array}$$ (1)

where fuzzy domain is represented by a, b and c values. Whereas, normal class is represented by x value of the extracted dataset before fuzzification process. The researchers are considered increasing rate of detection and reducing the false alarm rate that main motives of applying fuzzification data. It has the ability to resolves confusion and ambiguity via generating five values for each features that extracted from trace file. However, According to Eq 1, each extracted feature will paired with five values from the fuzzy domain. The proposed fuzzy model approves that [0, 1] value is range of the interval.

### C. Generate Sybil and Wormhole attacks

To test the security system, we need to determine two types of behaviours: normal and abnormal. The wormhole behaviour leads to forwarding received packets to channel communication or private paths between two vehicles rather than resending these to the destination vehicle or RSU in that zone. The attacks are simulated in ns-2.35RC7 [20] using the Object Tool Command Language (OTCL) script to some files in simulator. During abnormal behaviour some files are altered in the AODV routing protocol in order to create Sybil attack. The proposed IDS is based on features that were extracted from the trace file and routing file generated from ns-2 as well as distances obtained for each vehicle. This is achieved by using *X* axis, *Y* axis and *Z* axis coordinates from General Position System (GPS), which is a basic sensor that we assume each self-driving vehicle is equipped with.

The simulated highway scenario consists of 70 vehicles and six RSUs [20]. There are three Sybil vehicles and two Wormhole vehicles in this simulated scenario. The detection is based on behaviour of vehicles, such as Sybil attacks claiming multiple fake identities. At the same time the Wormhole attacks forward CAMs to private channels with other vehicles.

### D. Simulator environment and parameters

System simulators are common tools used for evaluating intrusion detection in ad hoc networks [20]. The performance and the reliability of proposed IDS are studied by simulators without using real vehicles. To assess the proposed IDS, we simulated VANETs by using ns-2 to evaluate the performance of the proposed IDS [13]. This was chosen owing to its rich library, free availability and open source. OTCL, TCL and AWK scripts were produced to analyse the data that have been generated from the trace file and routing table. The performance of the IDS is computed in terms of throughput, Packet Delivery Ratio (PDR) and packet delay. Table 2 gives the parameters of our simulation in ns-2.

**Table 2:** Simulation environment and parameters.

Parameters	Value
Simulation	ns2.35—RC7
Simulation Time	500s
Number of Nodes	70 Vehicles
Number of RSUs	4 RSU
Type of Traffic	Constant Bit Rate (CBR)
Topology	800*800 (m)
Transport Protocol	UDP
Packet Size	512
Routing Protocol	VAODV- VANETs
Channel Type	Wireless
Queue Length	50 packets
Number of Road Lanes	2
Radio Propagation Model	Two Ray Ground
MAC Protocol	IEEE 802.11 Ext
Number of Malicious Vehicles	2
Speed	75 m/s
Interface Queue Type	priority Queue
Network Interface Type	Physical Wireless
Mobility Models	Highway Mobility Model

### E. Assumption

Cooperative Awareness Messages (CAMs) reflect the condition of the surrounding environment and status messages of other vehicles that have joined the area surrounding the vehicle in radio range. The status messages contain important information such as curvature, position, speed, acceleration, weather, ID, and more. In VANETs communication, each self-driving vehicle acts as a router and host, as vehicles may spontaneously be added or removed from the VANET. Hence a vehicle may be source vehicle at time *t = 0* to generate CAMs. The same vehicle can function as destination to receive packets sent at time *t = n*. These packets may have been generated from other source vehicles and other intermediate vehicles between source and destination like relay vehicles.

We need to establish some rules to receive the CAMs otherwise they will be discarded. These rules make the performance of the proposed security system more efficient at detection rate by reducing the number of false alarms and make more efficient use of network resources such as bandwidth. These rules are [23]:

1. The current CAM must differ by at least 4 degrees in value of heading from the previous messages or
2. The current CAM must differ by at least 4m in position from the previous message or
3. The current CAM must differ by at least 0.5 m/s in speed from the previous message or
4. The current CAM must differ by at least 1s in time from the previous message.

To avoid channel congestion and increase amount of dropped packets, these rules have to be checked every 100ms [24] [25].

### F. Communication area

A self-driving vehicle that would like to communicate with RSUs or vehicles must be in cluster mode. In a clustering scheme, we need to identify just one vehicle to be the CH for the TDMA communication. When another self-driving vehicle joins a cluster area, the group must select one vehicle as CH to manage the group and control transfer of data between multiple vehicles also vehicles and RSUs. The success of this scheme depends on the existing cooperation between CMs and CHs, and this cooperation should be within the coverage area. In other words, the vehicles and CHs should be inside the transmission range (Tr) that help to report abnormal behaviour from vehicles to CH in that zone. The area of vehicle is calculated based on a formula given below in Eq 3 [9]:

$$\begin{array}{c} Area\left(Vr\right)=T_{r}\left(V_{r}\right)-T\left(S_{max}-S_{min}\right) \end{array}$$ (2)

where, Tr (Vr)—Transmission range of self-driving vehicle Vr. T—Packet latency in vehicles. *Smax*—Maximum vehicle’s speed while *Smin*—Minimum speed of vehicles.

The IDS algorithm relies on the following basic principles:

1. Malicious vehicles drop or duplicate the data or control that have been received from other surrounding vehicles. These vehicles try to create congestion in the network.
2. The normal behaviour of honest self-driving vehicle is to forward packets that have been received to the right destination.

### G. IDS parameters

The accuracy detection and false alarm rate depend on the number and type of parameters that are used while designing the detection scheme [9]. In our system, we used four types of parameters: the routing table, distance, timestamps and forward value *(Fv)*. To obtain these parameters, each vehicle must collect data from its neighbour vehicles in inter-clustering. The following parameters describe normal and abnormal behaviours of self-driving vehicles in VANETs:

### 1. Routing table

The routing table provides communication data of any vehicle whether in intra-clustering or inter-clustering of self-driving vehicles. In our proposed system, each vehicle has an IDS to sniff, analyse and identify normal / abnormal behaviours. To generate a routing table, we need to add a function in the routing protocol. Table 3 shows basic information of routing table generated in ns-2.

**Table 3:** Routing table.

Notation	Value
Vehicle ID	2
Current Time	1.00949
Destination ID	3
Next Hop	3
Number of Hops	1
Sequence Number	4
Expire Time	7.0009
Flags	1

The IDS on each vehicle will extract the following from routing table: vehicle ID, time and number of hops to detect Wormhole attacks.

### 2. Distance and angle of vehicles

A measure of distance between vehicles is an important factor in our proposed security system. Each self-driving vehicle in clustering mode can calculate distance between itself and other vehicles, based on values of x-axis and y-axis obtained from their GPS. The proposed system is based on Eqs 1 and 2 to calculate the distance and angle between two vehicles:

$$\begin{array}{c} Distance=\sqrt{\left[{\left(x_{2}-x_{1}\right)}^{2}+{\left(y_{2}-y_{1}\right)}^{2}\right]} \end{array}$$ (3)

$$\begin{array}{c} Angle=arctan\left(x_{2}-x_{1}\right)/\left(y_{2}-y_{1}\right) \end{array}$$ (4)

where: *(x1, y1)* is the position of the first vehicle and *(x2, y2)* is the position of the second vehicle.

### 3. Forward value

The forward value plays an important role in increasing the detection accuracy in self-driving vehicles. The IDS can calculate the forward value of each vehicle; it makes decisions based on the *Fv*. It is calculated from trace file that have been generated from ns-2. The IDS considers each vehicle to be malicious when the vehicle does not forward a received packet to the destination after a particular time *(T)* and the forward value *(Fv)* will be increased by 1 unit. In other words, it will increase by 1 every time abnormal behaviour is observed. The *Fv* is communicated to all neighbour vehicles and they update their stored value with the latest values. The proposed system considers the behaviour of vehicles as normal when the *Fv* is higher than the threshold (e.g. set to 3), otherwise the system will considered abnormal.

### H. Authentication phase

**Fig 5:**
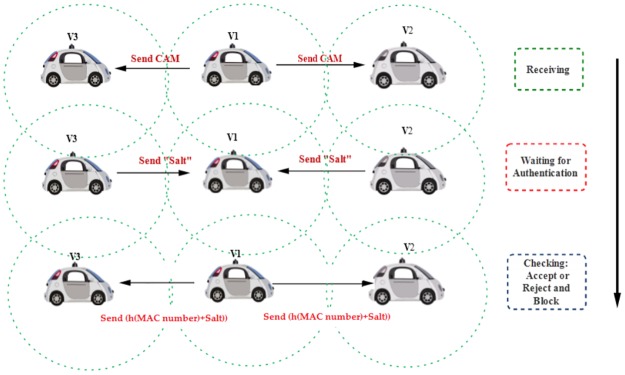
Authentication scenario.

In this paper, we proposed a novel authentication technique to protect the external communication system of autonomous vehicles. It is considered one of the most important security part which must be supported for each wireless communication system. The proposed authentication system has the ability to assist the self-driving vehicles to identify between authorised and unauthorised cars so that these vehicles can communicate with other vehicles and RSUs in that radio coverage area. In more details, the authentication process is heavily based on MAC number that has been generated from any device on the on board unit such as sensors, GPS or Lider. The authentication scenario is shown in Fig 5.

The security system in this paper assumes that each mobility vehicle has the hash value that extracted from the devices on board unit of self-driving vehicles. The hash value that generated from MAC number with salt value that is considered an identifying aspect for each autonomous vehicles on roads.

Definition: salt value is random number that integrated with messages from source vehicle to destination vehicle. This value plays important role in increase the protection of the communication system of autonomous vehicles.

The authentication scenario:

- The vehicle-V1 send message to the vehicles V2 and V3.
- The Vehicles V2 and V3 send salt number to the source vehicle and wait.
- The vehicle-V1 sends summation of the salt value with h(MAC) value to the destination vehicles (V2 and V3).
- The Vehicles V2 and V3 will match the received value with their own value. If the value matches the decision it “accepts the message”, otherwise they will “reject and block the communication with vehicle-V1”.

The algorithm below shows an algorithm vehicles authentication that proposed to protect the external communication for autonomous vehicles.

Vehicles Authentication Algorithm Input:

- Started when vehicles with in rang of central transmission.
- Authorised vehicles are understanding the MAC number.
- Central Vehilce = v1, client Vehicles = v2, v3, … vn.

Procedure:

- Vehicle V1 send CAM message to the fixed radio rang.
- Authorised vehicles (with rang) v2 … vn send response message (salt) and wait.
- Vehicle v1 send summation (salt + h(MAC)) to the destination vehicles.
- Destination vehicles will match the received value with own value.

Output:

- Accept communication if matching the received value.
- Otherwise, Reject communication.

End.

### I. Intelligent intrusion detection

**Fig 6:**
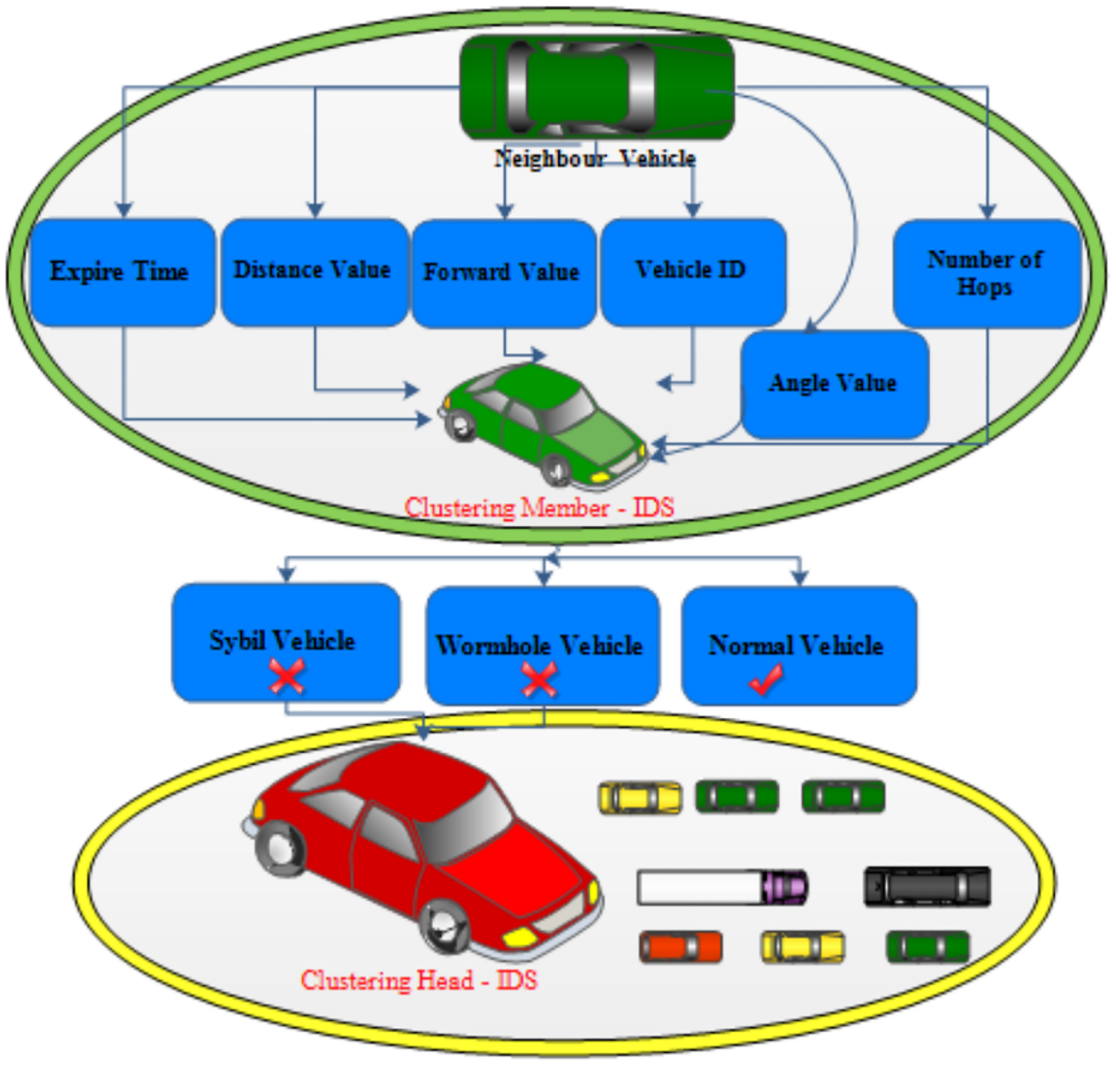
IDS architecture.

In the clustering scheme, we install IDS on each self-driving vehicle. The role of CMs is to collect information of neighbour vehicles in the zone. It is assumed that CHs are trusted in external communication of self-driving vehicles. Each vehicle uses rules and thresholds to detect abnormal behaviour when identifying a malicious vehicle. Whether Sybil or a Wormhole attack are detected; the vehicle will have sent a message to notify its CH. The CH will block and broadcast the malicious ID’s to its CMs and to other CHs. The following are the 7 stages of utilised, and the overall architecture of the proposed security system is shown in Fig 6.

1. Generate the highway mobility - In this stage, two tools are utilised to generate highway mobility and traffic to simulate the real communication environment of self-driving vehicles (see above). The output files of this stage are considered input files to ns-2 to generate trace file and routing table of normal and abnormal behaviour.
2. ns-2 - CMs will determine information from other vehicles. They generate a routing table for each vehicle. Each vehicle will broadcast 3-10 packets/second [10]. The CMs can extract features like timestamp, vehicle ID, GPS position and number of hops from routing table and trace file.
3. Distance and angle calculation - In this stage, the proposed system can calculate distance and angle degree between vehicles based on values of X-axis and Y-axis obtained from GPS and applied on Eqs 1 and 2.
4. Detection phase - In this stage, the IDS on CMs has ability to detect the wormhole attacks from parameters that have been extracted from the routing table and trace file. The parameters are: number of hops, forward value and time. The IDS on CMs can identify the Sybil vehicles from normal vehicles based on some important features such as distance, angle and vehicle ID.
5. CMs - The IDS on CMs will send notification to CH when it detects malicious behaviour. It sends warning message with full details about the malicious vehicle that is detected in clustering mode.
6. Reaction of CHs - The CH will generate alarms and block the malicious vehicle to alert other vehicles in the inter-cluster and sends the same warning message to all CHs and RSUs in that zone.
7. Performance metrics - In this stage, we evaluate the proposed IDS by calculating the performance metrics such as the packet delay rate, (PDR) and throughput.

As shown in Fig 6, the proposed system has seven parameters as input to CMs while it has three outputs: malicious vehicle (Sybil / wormhole) and normal vehicle.

## 6. Simulation results and analysis

**Fig 7:**
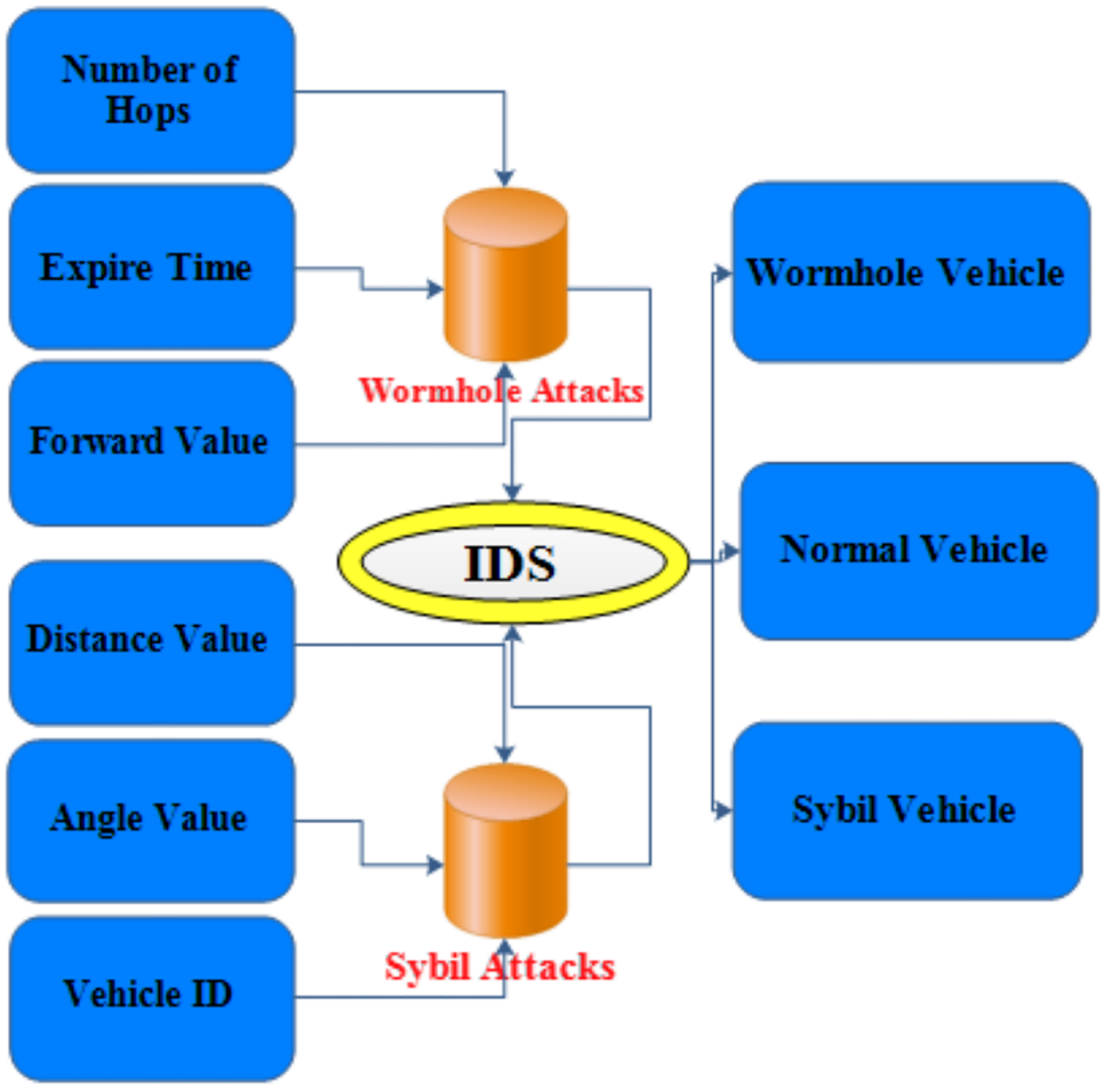
Type parameters of detection.

The IDS can detect two most common but serious attacks in VANETs: Sybil and Wormhole attacks. As we know each of these attacks has a different behaviour. Thus, each attack has different parameters to detect malicious behaviour. Fig 7 shows type parameters of proposed IDS.

Here, to evaluate the proposed IDS performance, we need to analysing efficiency, effectiveness, and calculate the performance metrics. First, Table 4 is generated which describes the different parameter values that have been extracted, calculated and stored by each vehicle.

**Table 4:** Shows some extracted and calculated parameters.

Time	Parameters	V1	V2	V3	V4	V2
T0	Vehicle-ID	V0	V1	Vworm	V5	V9sybil
T0	Destination Value	64.6m	97.7m	130m	67.2m	97.7m
T0	Angle Value	-50.6d	-30.7d	-21d	-48.01d	-30.7d
T0	Time Stamp	7s	7s	7s	7s	7s
T0	Forward Value	1	3	2	1	3
T0	Number of Hops	1	2	1	3	2
T1	Vehicle-ID	Vw	V1	V4	V5	V8sybil
T1	Destination Value	8.68m	139.2m	139m	107.3m	139.2m
T1	Angle Value	-35.1d	-21.03d	-21d	-27.7d	-21.03d
T1	Time Stamp	10s	10s	10s	10s	10s
T1	Forward Value	1	5	3	11	5
T1	Number of Hops	1	3	5	3	3
T2	Vehicle-ID	V0	V1	V4	V5	V6sybil
T2	Destination Value	126.3m	139m	139.2m	111.8m	139m
T2	Angle Value	-23.3d	-21d	-21d	-26.5d	-26
T2	Time Stamp	16s	16s	16s	16s	16s
T2	Forward Value	1	10	8	12	8
T2	Number of Hops	1	2	3	5	4

Table 4 demonstrates the sample database of vehicles that have been collected and calculated from routing table and trace file. According to Table 4, Sybil attacks are detected by using distance and angle. To detect the wormhole attacks, the forward value and number of hops are used. The simulation process is applied 20 times to evaluate/test of the proposed security and authentication systems in this research. In addition, the average classification rate is calculated in Table 5 of two types of attacks targeting autonomous vehicles in VANETs.

**Table 5:** Classification rate.

IDS	Accuracy	Class
IDS-Clustering	76.84	Normal
IDS-Clustering	95.7	Abnormal

**Fig 8:**
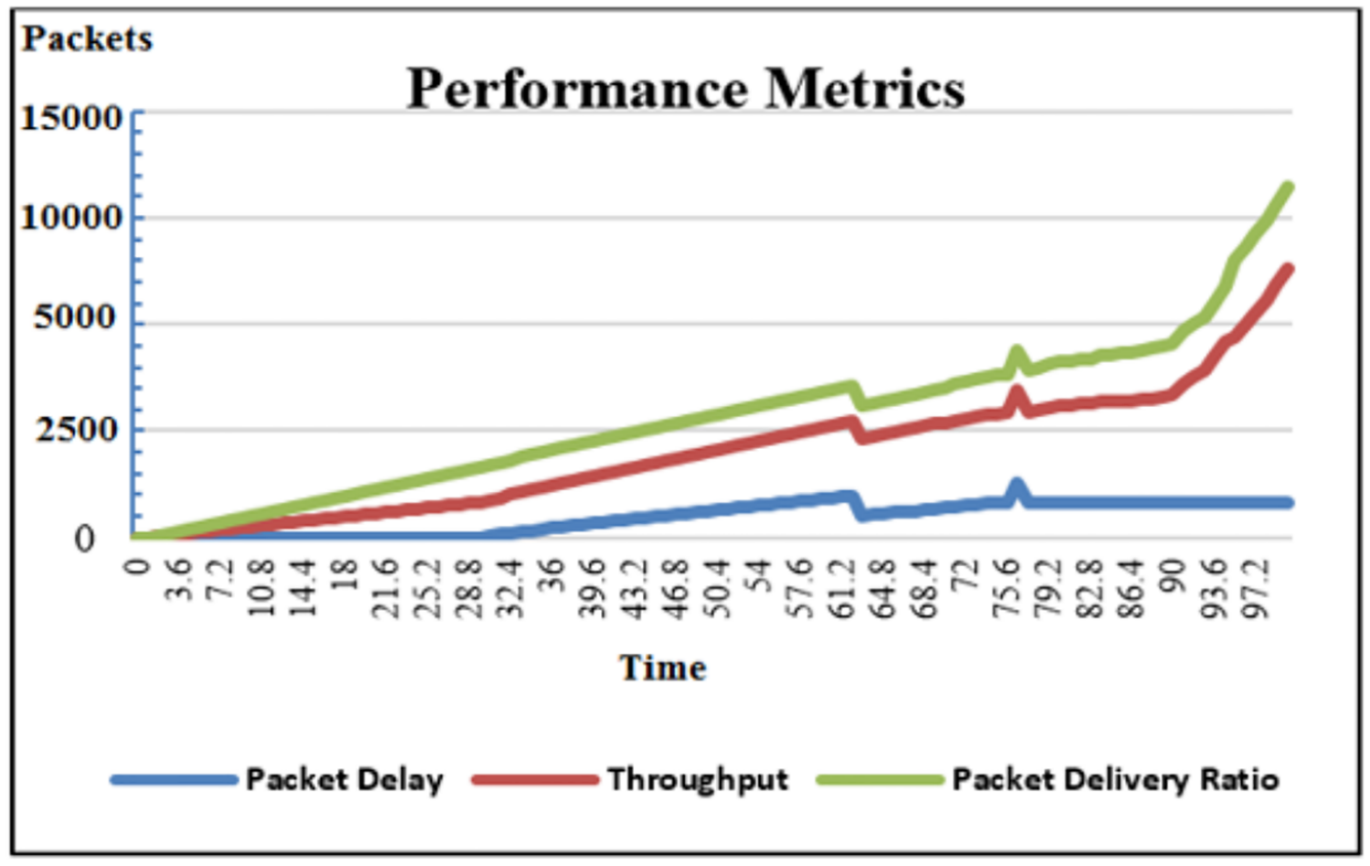
Performance metrics.

The efficiency of this IDS is assessed using ns-2 under two conditions: self-driving vehicles with IDS and self-driving vehicles without IDS. To evaluate the efficient of VANET with IDS, we calculate the performance metrics of VANETs, such as packet delivery ratio (PDR), packet delay and throughput [25], as shown in Fig 8:

It is easily notice from the simulation results that this IDS can play a vital role on enhancing the performance of the external communication in autonomous and semi-autonomous vehicles. The proposed security system can overcome one of the common security problems, which is due to the lack of fixed security infrastructures by using clustering mode in the external communication systems a virtual gateway of control. It is built on control data and sensitive information that sent/received between vehicles and their RSUs on the road side.

## 7. Discussion

The motivation of our work is to design a security system that protects external communications in self-driving and semi-autonomous vehicles. The proposed security system is implemented in seven stages namely:

- Generating the highway mobility model.
- network simulation to generate trace table and routing table.
- Calculate distance and angle.
- The detection phase.
- The role of CMs.
- The reaction of CHs.
- Performance metrics.

**Fig 9:**
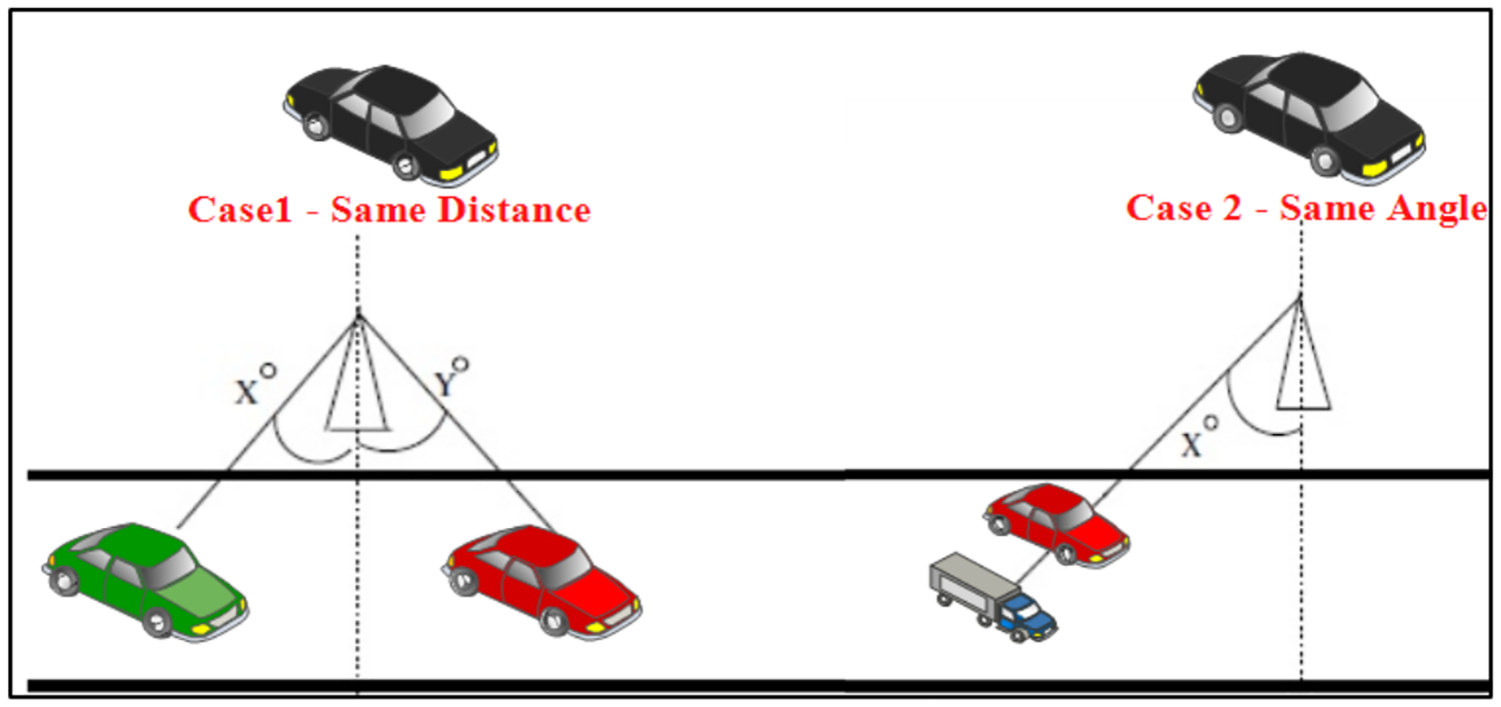
Case 1 and Case 2 of self-driving vehicles.

This security system can overcome two common problems which are that some self-driving vehicles have the same angle degree but different distances and others have the same distance but different angles degree. If the proposed IDS is just based on these features it will be confused in detection which would directly have a negative impact on detection rate and the number of false alarms, as shown in Fig 9.

In order to validate this system, we need to compare our results with other security system such as FPN-IDS [26].

From Table 6, it is easily noticed that the vital role of the IDS-clustering in enhancing detection rate of normal behaviour in self-driving vehicles, whereas IDS-FPN provides a better detection rate than IDS-clustering. In the futur ework, we could for example design clustering FPN to obtain better result on determining normal and abnormal behaviours.

**Table 6:** Classification rate.

IDS	Accuracy	Class
IDS-Clustering	76.84	Normal
IDS-Clustering	95.7	Abnormal
IDS-FPN	58.33	Normal
IDS-FPN	100	Abnormal

The design of hierarchical IDS based on clustering mode enhances the detection rate of proposed IDS in the external communication systems by 20.15 compared to Coussement and et al. [20]. Hence the IDS-clustering has a direct and positive impact on the resulting system because of the increase in the detection rate, decrease in the false alarm rate and error rate. The proposed system can be extended to build other IDS which can detect other types of attacks such as flooding, black hole and grey hole attacks.

Intelligent IDS is proposed in this paper to detect Sybil and Wormhole attacks that based on clustering mode. The detection process is heavily based on features that extracted from trace file and routing protocol. These files are generated from network simulator. Whereas, FPN-IDS is designed to detecting Flooding and Dropping attacks that targeted control data and sensitive information of vehicles [26]. The main aspect difference between FPN-IDS and Clustering-IDS is shown in Table 5 below.

According to Table 7, we can easily distinguish between two IDS. As a result, the IDS is proposed in this paper definitely different from FPN-IDS.

**Table 7:** Comparison between IDS-Clustering and FPN-IDS.

Features	Clustering-IDS	FPN-IDS
Detection tools	Clustering	Fuzzy Petri Net
IDS Architectural	Hierachal	Cooperative
Attacks Type	Sybil and Wormhole	Dropping and Flooding
Detection File Source	Trace File	Trace File and Routing Protocol
Routing Protocol	Vehicle AODV	Original AODV
Pre-precessing Dataset	Fuzzification dataset	Normal dataset

## 8. Conclusion and future work

This paper detailed the design of an IDS to efficiently detect malicious vehicles and enhance the performance of VANETs. An approach to detect Sybil and Wormhole attacks, which have an adverse effect on the communication and authenticity of self-driving vehicles. The designed IDS aims to develop an IDS that identifies and isolates malicious vehicles. Each CH uses the IDS to protect external communication of self-driving from malicious vehicles. They can compare the difference of various parameters obtained from different vehicles at regular intervals. Parameters like distance, angle degree, forward value and number of hops play a crucial role in detection Sybil and Wormhole attacks in VANETs. The distance and angle degree are unique parameters, obtained from each mobile vehicle at any time. Moreover, if the distance between two vehicles is same, the angle degree of these vehicles differentiates the normal and malicious vehicles. The number of vehicles has an important role in increasing the detection time of abnormal behaviour in the external communication systems of these vehicles.

Our proposed system has the ability to detect Sybil and Wormhole attacks by monitoring/ analysing the routing table and trace file that have been generated from the network simulator. The trace file describes the behaviour of the network through the send, receive, move, forward and drop packets. A possible further extension of the system is to design IDS on RSUs as well as designing IDS with Artificial Intelligent (AI) techniques such as neural networks and k-nearest neighbour.

Future trends of driverless cars are concentrated the safety of the overall traffic environment. The Institute of Electrical and Electronics Engineers (IEEE) predicts that driverless cars will account for up to 75 percent of vehicles on the road by the year 2040. No driving equals more safety because there’ll be no more text or drunk driving. In addition, cars without drivers are smart enough to avoid accidents. There must be enough sensor technology, enough computing power within the automotive and computer algorithms that detect the data output of sensor images, real-life traffic situations and give good feedback to the car. This is the technical challenge, and the manufacturers are working hard to generate new smart environment.

## Supporting information

S1 File This dataset is generated by the network simulation ns-2 [27]. (RAR)
